# Parental History of Hypertension: A Risk for Autonomic Dysfunction and Metabolic and Vascular Derangement in Normotensive Male Offspring

**DOI:** 10.7759/cureus.44636

**Published:** 2023-09-04

**Authors:** Charu Bansal, Saranya Kuppusamy, Senthil Kumar Gandhipuram Periyasamy, Harichandrakumar KT, Jean Fredrick, Senthil Kumar Subramanian

**Affiliations:** 1 Physiology, Jawaharlal Institute of Postgraduate Medical Education and Research, Puducherry, IND; 2 Biochemistry, Jawaharlal Institute of Postgraduate Medical Education and Research, Puducherry, IND; 3 Biostatistics, Jawaharlal Institute of Postgraduate Medical Education and Research, Puducherry, IND; 4 Physiology, All India Institute of Medical Sciences, Kalyani, IND; 5 Physiology, All India Institute of Medical Sciences, Madurai, IND

**Keywords:** relaxin, apelin, metabolic profile, baroreflex, cardiac autonomic dysfunction, parental history of hypertension

## Abstract

Background: Children of hypertensive parents have an increased propensity of developing hypertension, at an age very much prior to their parents. Understanding the pathophysiology of hypertension in such young individuals, especially baroreflex sensitivity (BRS), is necessary. Reduced heart rate variability (HRV), insulin resistance (IR), dyslipidemia, and decreased vasodilatory adipokines, namely, apelin and relaxin, in normotensives may predispose to the onset of hypertension. Thus, this study compared autonomic functions, vascular markers, and metabolic profiles between normotensive male offspring with and without parental hypertension.

Methods: This analytical cross-sectional study comprised 40 male normotensive offspring of hypertensive parents, aged 18-35 years, recruited as the study group and 40 age- and body mass index (BMI)-matched normotensive male offspring with non-hypertensive parents enrolled as controls. Cardiovascular autonomic functions, including BRS, HRV, diastolic blood pressure response to isometric handgrip test (ΔDBPIHG), Valsalva ratio, and metabolic and vascular markers, were assessed.

Results: The study group exhibited reduced BRS, HRV, and Valsalva ratio and higher ΔDBPIHG compared to controls, indicating impaired autonomic functions. The study group had higher IR and triglyceride levels and reduced apelin and relaxin levels. BRS showed significant correlations with HRV, Valsalva ratio, ΔDBPIHG, and metabolic and vascular markers.

Conclusions: Normotensive male offspring of hypertensive parents exhibit impaired autonomic functions, as evidenced by reduced BRS, HRV, and Valsalva ratio. Additionally, they have higher IR, dyslipidemia, and decreased levels of vasodilatory adipokines, indicating an increased risk for future hypertension development. These findings signify that early identification of hypertensive potential in this high-risk population is warranted, which would enable taking necessary preventive measures.

## Introduction

Hypertension affects nearly one-third of Indians [1]. Prominent among the non-modifiable risk factors for hypertension is a family history of the condition, which encompasses other factors such as age over 65 years and the presence of coexisting diseases such as diabetes or kidney disease. The offspring of hypertensive parents are particularly vulnerable, as they are more prone to become hypertensives, at a younger age than their parents [2].

Understanding the pathophysiology of hypertension in this vulnerable population is of utmost importance to provide early intervention and preventive measures. Autonomic dysfunction, characterized by increased adrenergic activity and decreased vagal drive, has long been implicated in the pathogenesis of essential hypertension [3]. This dysfunction ultimately leads to alterations in the baroreflex response, a crucial mechanism controlling blood pressure (BP) in the short term.

Furthermore, the renin‐angiotensin‐aldosterone system has had a crucial association with long-term BP regulation. Recent research has highlighted the modulatory effect of baroreceptors on BP over extended periods [4]. Baroreflex sensitivity (BRS), an index of baroreflex-mediated BP regulation, can be measured at rest, in response to pharmacological agents, or during the Valsalva maneuver (VM). BRS assessment during the VM offers the advantage of quantifying BRS across a broader range in a short period [5].

Altered BRS has emerged as a pivotal factor in the onset of hypertension. Studies have reported that normotensive individuals with a parental history of hypertension, particularly young females, exhibit lower BRS, indicating a more pronounced reduction in BRS in the offspring of hypertensive parents [6]. Given that males have a higher predisposition to cardiovascular diseases (CVDs) than females, it becomes essential to evaluate male offspring of hypertensive parents as well [7].

Previous research conducted on male offspring of hypertensive parents has demonstrated reduced BRS, indicating their hypertensive potential [8]. Additionally, resting autonomic tone, assessed through heart rate variability (HRV), was reported as diminished in normotensive children with hypertensive parents [9]. These individuals also exhibit exaggerated diastolic blood pressure (DBP) responses to isometric handgrip, suggesting concealed hypertensive potential and underlying exaggerated sympathetic reactivity. Reduced BRS has been proposed as a contributing mechanism for this excessive vascular reactivity [10,11].

Interestingly, normotensive children of hypertensive parents have been reported to exhibit hyposecretion of vasodilatory adipokines, such as apelin and relaxin, which may further contribute to the pathophysiology of hypertension in this population [12].

Moreover, autonomic dysfunction and metabolic derangements share a causal inverse relationship [13]. BRS has been found to be inversely related to insulin resistance (IR), even in seemingly healthy individuals [14]. Hyperinsulinemia, independent of insulin resistance, has also been suggested to lead to dysfunctional BRS [15]. Furthermore, altered lipid metabolism has been observed in young normotensive offspring with a family history of hypertension, and dyslipidemia has been linked to decreased BRS and increased blood pressure [16,17].

Considering the potential interplay between autonomic imbalance, metabolic abnormalities, and altered vasodilatory adipokine levels, it becomes essential to investigate their association with BP regulation in normotensive male offspring with hypertensive parents. This study aims to extend our understanding of these relationships, providing valuable insights into the pathophysiology of hypertension and enabling the design of early preventive measures.

## Materials and methods

Study design and setting

This analytical cross-sectional study was carried out at the Department of Physiology, Jawaharlal Institute of Postgraduate Medical Education and Research, Puducherry, India, following approval from the Institute Human Ethics Committee (JIP/IEC/2021/030).

Study participants

Healthy male participants aged between 18 and 35 years were recruited. Participants with hypertensive parents (both or either) were enrolled in the study group, while participants with non-hypertensive parents were recruited as controls. Exclusion criteria included hypertension, cardiac disease, ocular diseases (retinopathy, glaucoma, and lens implantation), diabetes mellitus, recent surgery, and the use of medications that alter autonomic functions. Additionally, individuals whose parents had diabetes and other metabolic diseases were excluded from the control group.

Procedure

The study was conducted in the autonomic function testing laboratory, Department of Physiology, Jawaharlal Institute of Postgraduate Medical Education and Research. Written informed consent from the participants was obtained after thoroughly explaining the procedure. Participants were instructed to arrive at the laboratory at 9 am after an overnight fast, and the following parameters were measured.

Parameters measured

Demographic Details

Demographic details, including dietary patterns and physical activity levels, were collected. Physical activity was evaluated using the International Physical Activity Questionnaire-short (IPAQ short) [18]. A family history of hypertension, diabetes, and other cardiovascular diseases was documented.

Anthropometric Measurements

Height, weight, and waist and hip circumference were measured following standard procedures from the International Society for the Advancement of Kinanthropometry (ISAK) [19]. Height was measured using a stadiometer (BHH6, Easy Care, Mumbai, India), and body weight was measured using a digital weighing machine (MS 4900, Charder Electronics Co. Ltd, Taichung, Taiwan). Body mass index (BMI) was calculated using Quetelet’s index formula (BMI = weight (kg) / (height (m))^2^).

Basal Cardiovascular Measures

Following supine rest for 10 minutes, basal heart rate (BHR), systolic blood pressure (SBP), and diastolic blood pressure (DBP) were measured using a digital BP monitor (Omron, HEM-8712, Omron Healthcare Co. Ltd., Kyoto, Japan).

Cardiovascular Autonomic Function

Baroreflex sensitivity (BRS) during rest and VM: BRS was evaluated both at rest and while performing the Valsalva maneuver (VM). Continuous non-invasive BP and heart rate were measured using Finometer Pro (Finapres Medical Systems (FMS), Amsterdam, Netherlands). The computation and analysis for BRS were performed using the BeatScope® software (Finapres Medical Systems (FMS), Amsterdam, Netherlands) [20,21]. Subsequent to connecting the appropriate finger pressure and brachial pressure cuff, and with necessary calibration and height corrections, as per standard protocol, the subjects underwent 10 minutes of supine rest. Continuous BP and finger pulse waves were recorded for 10 minutes, and BRS during rest (BRSR) was computed using the BeatScope software. Then, the recording was continued, and the participants were asked to perform the Valsalva maneuver, which involved forced expiration after deep inspiration against the closed glottis, with closed nostrils, into a mouthpiece having air leak connected with a sphygmomanometer. During the maneuver, they were asked to sustain an expiratory pressure of 40 mmHg for 15 seconds. Lead II electrocardiogram (ECG) along with continuous non-invasive BP were measured during VM until one minute after completion. The BRS was estimated during phase II (phase of sustained strain, BRSVMII) and during phase IV (phase of recovery, BRSVMIV). Following this, five minutes of rest was given.

Heart rate variability (HRV): Short-term measurement of HRV was done using a lead II ECG [22]. Five minutes of resting lead II ECG was recorded using the BIOPAC-MP150 data acquisition system (BIOPAC, Goleta, CA, USA) with AcqKnowledge software version 4.2. Kubios HRV standard software version 3.2. (Kubios Oy, Kuopio, Finland) was utilized for HRV analysis. Time domain indices (square root of the mean of the sum of the squares of differences between adjacent NN intervals (RMSSD), standard deviation of all NN intervals (SDNN), number of pairs of adjacent NN intervals differing by more than 50 ms in entire recording (NN50), and percentage of NN50 (pNN50)) and frequency domain indices (very low frequency (VLF) (ms^2^), low frequency (LF) (ms^2^), high frequency (HF) (ms^2^), total power (TP) (ms^2^), LF power in normalized units (LFnu), HF power in normalized units (HFnu), and LF/HF ratio) were computed.

BP response to isometric handgrip test: DBP during isometric hand grip (IHG) was measured at one-third of the maximum and sustained voluntary capacity for three minutes. The change of DBP during the maneuver (∆DBPIHG) (the difference between the maximum DBP attained throughout the handgrip procedure and baseline DBP) was determined.

Valsalva ratio: The Valsalva ratio was calculated during VM by dividing the lengthiest RR interval of phase IV by the shortest RR interval of phase II.

Biochemical Parameters

Blood (5 mL) (after 12 hours of overnight fasting) was collected under aseptic precautions for biochemical analysis. Fasting serum glucose and lipid profile assay were done on the same day. Serum (2 mL) was stored at -80°C for apelin, relaxin, and insulin assay. Glucose and lipid profiles (total cholesterol, triglyceride, very low-density lipoprotein (VLDL), low-density lipoprotein (LDL), and high-density lipoprotein (HDL)) were assayed in autoanalyzer AU 5811 (Beckman Coulter, CA, USA). Serum insulin level was measured using a diagnostic kit by Calbiotech (EI Cajon, CA, USA) based on the solid phase sandwich ELISA method. Insulin resistance was estimated using a homeostatic model assessment of insulin resistance (HOMA-IR), as “HOMA-IR = fasting plasma glucose (mg/dL) × plasma insulin (µIU/mL) / 405.” Apelin and relaxin levels were measured using diagnostic kits from Abbkine (Wuhan, Hubei, China), based on a two-site sandwich ELISA.

Sample size estimation

The sample size was calculated with an expected difference in mean BRS of 5 ms/mmHg between the groups and a standard deviation of 8 at a significance level of 5% with a power of 80% [6]. The method used to estimate sample size was a comparison of two independent means.

Statistical analysis

Data analysis was done using Statistical Package for the Social Sciences (SPSS) version 25.0 (IBM Corp., Armonk, NY, USA). Categorical data were expressed as percentages/frequencies, and intergroup comparison was done using chi-square or Fisher’s exact test. The normality of quantitative data was evaluated using the Shapiro-Wilk test. Quantitative data were expressed as mean (standard deviation (SD)/median (interquartile range)), and intergroup comparison was done using unpaired Student’s t-test or the Mann-Whitney U test based on data distribution. For association testing, Pearson’s or Spearman’s tests were applied based on data distribution. Regression analysis was performed to evaluate the independent variables related to BRS. All statistical analyses were done at a 5% significance level.

## Results

Among the study participants with parental hypertension, about 45% of the subjects had a history of maternal hypertension, around 47.5% had a history of paternal hypertension, and 7.5% had a history of both maternal and paternal hypertension. Dietary behavior and physical activity were comparable among the groups, with no significant difference.

Anthropometric and basal cardiovascular parameters are represented in Table 1. Age, weight, height, BMI, hip circumference, and waist/hip ratio were comparable among the groups. Waist circumference and waist/height ratio were increased significantly in the study group. Basal heart rate, SBP, DBP, and mean arterial pressure (MAP) were found to be significantly higher among study participants. The values were within the normal range in both groups.

**Table 1:** Comparison of anthropometric and baseline cardiovascular parameters between the control and study group The values are expressed in median (interquartile range). The Mann-Whitney U test was used to compare the median difference between the two groups. ^†^The value is expressed in mean±SD. Independent Student’s t-test was used to compare the mean difference between the two groups. *P<0.05 is considered statistically significant. SD: standard deviation

Anthropometric and basal parameters	Control group (n=40)	Study group (n=40)
Age (years)	23.00 (4.25)	23.00 (6.25)
Weight (kg)	71.00 (9.50)	72.35 (18.25)
Height (m)	1.70 (0.13)	1.71 (0.11)
Waist circumference (m)^†^	0.86±0.06	0.89±0.07^*^
Hip circumference (m)^†^	0.99±0.07	1.00±0.12
Body mass index (kg/m^2^)^†^	24.11±1.94	24.73±3.59
Waist/hip ratio	0.87 (0.07)	0.90 (0.04)
Waist/height ratio	0.50 (0.06)	0.53 (0.07)^*^
Heart rate (bpm)^†^	68.58±8.33	74.55±10.50^*^
Systolic blood pressure (mmHg)	110.50 (10.0)	116 (7.50)^*^
Diastolic blood pressure (mmHg)	65 (10.25)	69 (7.50)^*^
Mean arterial pressure (mmHg)	80.83 (6.92)	83.67 (5.83)^*^
Pulse pressure (mmHg)	45.78±7.43	47.83±7.64

From HRV analysis, time domain parameters such as SDNN and RMSSD were significantly decreased in study participants. Frequency domain parameters, namely, total power (TP), HF power, and HFnu, were significantly diminished among the study group. LFnu and LF/HF ratio were observed to be significantly elevated among study participants. Valsalva ratio was observed to be reduced significantly, while ΔDBPIHG was increased significantly among the study group. BRSR, BRSVMII, and BRSVMIV were significantly diminished among the study group compared to controls (Table 2).

**Table 2:** Comparison of cardiovascular autonomic parameters between the control and study group The values are expressed in median (interquartile range). The Mann-Whitney U test was used to compare the median difference between the two groups. ^†^The values are expressed in mean±SD. Independent Student’s t-test was used to compare mean differences between the two groups. *P<0.05 is considered statistically significant. HRV: heart rate variability, BRS: baroreflex sensitivity, SD: standard deviation, SDNN: standard deviation of all NN intervals, RMSSD: square root of the mean of the sum of the squares of differences between adjacent NN intervals, NN50: number of pairs of adjacent NN intervals differing by more than 50 ms in the entire recording, pNN50: percentage of NN50, LF: power in the low-frequency range (0.04-0.15 Hz), LFnu: LF power in normalized units, HF: power in the high-frequency range (0.15-0.4 Hz), HFnu: HF power in normalized units, TP: total power, LF/HF ratio: ratio of LF (ms^2^)/HF (ms^2^), ΔDBPIHG: change in diastolic blood pressure during isometric hand grip test, BRSR: baroreflex sensitivity at rest, BRSVMII: baroreflex sensitivity at Valsalva phase II, BRSVMIV: baroreflex sensitivity at Valsalva phase IV

Parameters	Control group (n=40)	Study group (n=40)
Short-term HRV (time domain indices)		
SDNN (ms)^† ^	57.67±21.86	45.90±17.12^*^
RMSSD (ms)	51.50 (34.33)	37.60 (25.13)
NN50 (count)	92.50 (110.50)	45.50 (73.25)
pNN50 (%)	25.45 (37.13)	12.85 (26.35)
Short-term HRV (frequency domain indices)		
TP (ms^2^)	3087.50 (3372.25)	2137 (1642.50)^*^
VLF (ms^2^)	990.50 (1204.75)	770 (743.50)
LF (ms^2^)	735.50 (806)	672 (645.50)
HF (ms^2^)	1043 (1146)	535 (543.25)^*^
LFnu^†^	40.20±18.32	52.46±10.16^*^
HFnu^†^	59.80±18.32	47.54±10.16^*^
LF/HF ratio	0.68 (0.81)	1.02 (0.57)^*^
Valsalva maneuver		
Valsalva ratio^†^	1.68±0.22	1.57±0.26^*^
Diastolic blood pressure response to isometric handgrip		
ΔDBPIHG (mmHg)^†^	16.38±5.60	20.43±4.70^*^
BRS		
BRSR(ms/mmHg)	18.72 (5.98)	14.53 (6.43)^*^
BRSVMII(ms/mmHg)^†^	9.68±3.02	7.48±3.13^*^
BRSVMIV (ms/mmHg)	21.25 (8.74)	17.15 (7.55)^*^

Metabolic profiles including insulin and HOMA-IR were significantly accentuated in the study group. Triglyceride (TG) and lipid risk ratios, namely, TC/HDL, LDL/HDL, and TG/HDL, and atherogenic index were significantly higher in the study group. Vascular parameters, i.e., serum apelin and relaxin levels, were reduced significantly among study participants (Table 3).

**Table 3:** Comparison of metabolic and vascular parameters between the control and study group The values are expressed in median (interquartile range). The Mann-Whitney U test was used to compare the median difference between the two groups. ^†^The values are expressed in mean±SD. Independent Student’s t-test was used to compare mean differences between the two groups. *P<0.05 is considered statistically significant. SD: standard deviation, FSG: fasting serum glucose, HOMA-IR: homeostasis model assessment-estimated insulin resistance, TC: total cholesterol, TG: triglycerides, HDL: high-density lipoprotein, LDL: low-density lipoprotein, VLDL: very-low-density lipoprotein Normal ranges of biochemical parameters: FSG: 70-100 mg/dL, insulin: <25 µIU/mL, HOMA-IR: <2.5; TC: <200 mg/dL, TG: <150 mg/dL, HDL: >40 mg/dL, LDL: <100 mg/dL, VLDL: <30 mg/dL

Parameters	Control group (n=40)	Study group (n=40)
Serum glucose and insulin		
FSG (mg/dL)^†^	81.43±5.17	83.23±3.32
Insulin (µIU/ml)^†^	5.55±1.01	8.17±1.51^*^
HOMA-IR^†^	1.11±0.20	1.68±0.32^*^
Lipid profile		
TC (mg/dL)^†^ (normal range : 70-100 mg/dL)	187.93±27.02	199.33±29.27
TG (mg/dL)^†^	115.30±27.75	130.93±34.73^*^
HDL (mg/dL)^†^	45.03±7.90	42.63±7.67
LDL (mg/dL)	111.50 (25.75)	120.50 (36.75)
VLDL (mg/dL)	20.00 (9.25)	19.50 (12.25)
Lipid risk ratio		
TC/HDL	4.20 (0.86)	4.67 (0.91)^*^
LDL/HDL	2.55 (0.65)	2.90 (0.84)^*^
TG/HDL	2.38 (1.03)	2.93 (1.35)^*^
Atherogenic index^†^	0.40±0.14	0.48±0.13^*^
Vascular parameters		
Apelin (ng/L)	1808.29 (1798.10)	1311.34 (858.37)^*^
Relaxin (pg/mL)	310.88 (346.78)	173.17 (152.82)^*^

BRSR had a significant negative correlation with waist circumference, waist/hip ratio, and waist/height ratio. No significant correlation was observed between BRSVMII and anthropometric parameters. BRSVMIV had a significant negative correlation with BMI, waist circumference, waist/hip ratio, and waist/height ratio.

BRSR and BRSVMIV had a significant negative correlation with HR; no significant correlation was found with SBP and DBP. No significant correlation was found between BRSVMII and basal cardiovascular parameters. BRSVMIV had a significant negative correlation with HR, and no significant correlation was found with SBP and DBP. BRSR showed a positive correlation with SDNN, RMSSD, TP, and HFnu, and a negative correlation was seen with LFnu and LF/HF ratio, all being significant. BRSVMII had no significant correlation with HRV indices. BRSVMIV had a positive correlation with SDNN, RMSSD, and HFnu, and a significant negative correlation with LFnu and LF/HF ratio, all being significant. BRSR and BRSVMIV had a significant positive correlation with Valsalva ratio and a significant negative correlation with ΔDBPIHG. BRSVMII had a significant negative correlation only with ΔDBPIHG.

BRSR had a negative correlation with parameters such as insulin, HOMA-IR, TC, TC/HDL, LDL/HDL, TG/HDL, and atherogenic index. BRSVMII had a significant correlation with LDL. BRSVMIV had a significant negative correlation with fasting serum glucose, insulin, HOMA-IR, TC, TC/HDL, TG/HDL, and atherogenic index. BRSR and BRSVMIV had a positive correlation with serum apelin and serum relaxin levels. BRSVMII had no correlation with vascular markers (Table 4).

**Table 4:** Correlation of BRS with anthropometric, cardiovascular autonomic, metabolic, and vascular parameters Correlation analysis using Spearman’s rank (r) correlation/Pearson’s correlation (r). P<0.05 is considered statistically significant. BMI: body mass index, WHR: waist/hip ratio, BHR: basal heart rate, SDNN: standard deviation of all NN intervals, RMSSD: square root of the mean of the sum of the squares of differences between adjacent NN intervals, TP: total power, LFnu: LF power in normalized units, HFnu: HF power in normalized units, ΔDBPIHG: change in diastolic blood pressure during isometric hand grip test, FSG: fasting serum glucose, HOMA-IR: homeostasis model assessment-estimated insulin resistance, TC: total cholesterol, TG: triglycerides, HDL: high-density lipoprotein, LDL: low-density lipoprotein, BRSR: baroreflex sensitivity at rest; BRSVMII: baroreflex sensitivity at Valsalva phase II, BRSVMIV: baroreflex sensitivity at Valsalva phase IV

Parameters	Baroreflex sensitivity (ms/mmHg) (n=80)		
	BRSR	BRSVMII	BRSVMIV
BMI (kg/m^2^)	r=-0.128	r=0.091	r=-0.228^*^
Waist circumference (m)	r=-0.334^*^	r=-0.209	r=-0.419^*^
Waist/hip ratio	r=-0.345^*^	r=-0.141	r=-0.368^*^
Waist/height ratio	r=-0.400^*^	r=-0.142	r=-0.450^*^
BHR (bpm)	r=-0.346^*^	r=-0.205	r=-0.288^*^
SDNN (ms)	r=0.328^*^	r=0.122	r=0.266^*^
RMSSD (ms)	r=0.468^*^	r=0.083	r=0.452^*^
TP (ms^2^)	r=0.307^*^	r=0.175	r=0.151
LFnu	r=-0.483^*^	r=-0.007	r=-0.523^*^
HFnu	r=0.483^*^	r=0.007	r=0.523^*^
LF/HF ratio	r=-0.483^*^	r=-0.012	r=-0.523^*^
Valsalva ratio	r=0.560^*^	r=0.176	r=0.448^*^
ΔDBPIHG (mmHg)^†^	r=-0.508^*^	r=-0.278^*^	r=-0.610^*^
FSG (mg/dL)	r=-0.155	r=-0.077	r=-0.263^*^
Insulin (µIU/mL)	r=-0.421^*^	r=-0.200	r=-0.281^*^
HOMA-IR	r=-0.457^*^	r=-0.179	r=-0.340^*^
TC (mg/dL)	r=-0.255^*^	r=-0.031	r=-0.447^*^
LDL (mg/dL)	r=-0.099	r=-0.221^*^	r=0.081
TC/HDL	r=-0.380^*^	r=-0.089	r=-0.496^*^
LDL/HDL	r=-0.266^*^	r=-0.185	r=-0.114
TG/HDL	r=-0.280^*^	r=-0.027	r=-0.376^*^
Atherogenic index	r=-0.280^*^	r=-0.050	r=-0.376^*^
Apelin (ng/L)	r=0.604^*^	r=0.027	r=0.579^*^
Relaxin (pg/mL)	r=0.658^*^	r=0.156	r=0.656^*^

Multiple linear regression further revealed the association between correlated parameters with BRSR, BRSVMII, and BRSVMIV as a dependent variable (Table 5).

**Table 5:** Multiple regression of BRS with cardiovascular autonomic and vascular markers Multiple linear regression analysis was done between autonomic and vascular parameters and BRS. *P<0.05 is considered statistically significant. BRS: baroreflex sensitivity, ΔDBPIHG: change in diastolic blood pressure during isometric hand grip test, BRSR: baroreflex sensitivity at rest, BRSVMII: baroreflex sensitivity at Valsalva phase II, BRSVMIV: baroreflex sensitivity at Valsalva phase IV

Variables	Regression coefficient B	95% confidence interval	
		Lower bound	Upper bound
BRSR(ms/mmHg)			
Valsalva ratio	9.072^*^	4.598	13.545
Apelin (ng/L)	0.001^*^	0.000	0.002
BRSVMII(ms/mmHg)			
ΔDBPIHG(mmHg)	-0.170^*^	-0.295	-0.044
BRSVMIV (ms/mmHg)			
ΔDBP (mmHg)	-0.342^*^	-0.615	-0.070
Relaxin (pg/mL)	0.011^*^	0.005	0.018

Valsalva ratio and apelin were observed to be significant independent contributors in the model for BRSR. It was estimated that with a one-unit increase in the Valsalva ratio, there was an average rise of 9.072 (95% CI: 4.598, 13.545) in BRSR. In the case of apelin, with every one-unit increase, there was an average rise of 0.001 (95% CI: 0.001, 0.002) units in BRSR.

The multiple regression model was tested with ΔDBPIHG and LDL as independent variables in the model for BRSVMII. It was estimated that with a one-unit increase in ΔDBPIHG, there was a decrease of 0.170 units (95% CI: -0.295, -0.044; P=0.009) in the BRSVMII. LDL did not show any significant contribution as an independent variable to BRSVMII.

In the model for BRSVMIV, ΔDBPIHG and relaxin were found to be significant independent contributors to BRSVMIV. It was estimated that with a one-unit increase in ΔDBPIHG, there was an average decrease of 0.342 units (95% CI: -0.615, -0.070) in the BRSVMIV. Similarly, with a one-unit increase in relaxin, there was an increase of 0.011 units (95% CI: -0.005, 0.018) in the BRSVMIV. The other independent variables did not show any significant contribution to BRSVMIV.

## Discussion

Hypertension is prevalent in India, leading to increased cardiovascular morbidity and mortality [1]. Parental hypertension is an independent risk factor for developing hypertension at an earlier age [2]. Understanding hypertension’s pathophysiology and blood pressure control mechanisms in vulnerable populations, especially high-risk males, is crucial. Our previous study reported decreased baroreflex sensitivity (BRS) in male offspring of hypertensive parents [9]. This study aimed at exploring the association between autonomic modulation controlling blood pressure and metabolic/vascular markers, potentially increasing hypertension risk in normotensive male offspring with/without parental hypertension history.

Our results indicated that waist circumference and waist/height ratio were significantly higher among offspring with hypertensive parents. As lifestyle patterns, including dietary behavior and physical activity, remained similar in both groups, hereditary influence may explain the difference observed in waist circumference and waist/height ratio. This is consistent with findings from other studies reporting increased BMI and central obesity in individuals with a familial history of hypertension [2].

Cardiovascular parameters, including basal heart rate, SBP, DBP, and MAP, were higher (within normal limits) in the children of hypertensive parents, supporting the presence of increased sympathetic activity in this group [23,24]. HRV analysis revealed diminished parasympathetic activity and heightened sympathetic activity, further confirming the autonomic imbalance in these individuals [22]. The exaggerated diastolic blood pressure response to the isometric handgrip procedure also indicated higher sympathetic reactivity to stress [10].

Baroreflex sensitivity, which plays a vital role in blood pressure regulation, was significantly reduced at rest and during phases II and IV of VM in the offspring with hypertensive parents. This reduction in BRS proposes a hypertensive potential and may be associated with sustained sympathetic overactivity [4]. Additionally, reduced BRS was correlated with waist circumference, insulin resistance, hyperinsulinemia, dyslipidemia, and reduced levels of apelin and relaxin. These factors collectively contribute to the sympathovagal imbalance and decreased BRS observed in these individuals.

The metabolic alterations observed in the offspring with hypertensive parents, such as increased serum insulin level, IR, and dyslipidemia, have been associated with increased sympathetic activity [25]. Dyslipidemia, specifically elevated triglyceride levels and altered lipid risk ratios, can contribute to reduced BRS and increase the risk of hypertension [17,26,27]. Furthermore, reduced levels of vasodilatory adipokines, apelin, and relaxin can lead to increased vascular tone and hypertension [12]. The reduced levels of key vasodilatory adipokines, specifically apelin and relaxin, play a critical role in modulating vascular tone, potentially leading to hypertension. Apelin, an endogenous peptide, offers diverse cardiovascular benefits, including vasodilation and blood pressure regulation. Decreased apelin levels relate to compromised endothelial function and impaired vasodilation. Similarly, relaxin, a peptide hormone in circulatory homeostasis, induces vasodilation via endothelium-dependent and endothelium-independent pathways. Lower relaxin levels can contribute to vascular dysfunction, fostering an environment for elevated vascular resistance and blood pressure. The interplay of reduced apelin and relaxin levels may significantly contribute to heightened vascular tone and hypertension, emphasizing their potential as therapeutic targets for vascular health management.

The reduced BRSR, BRSVMII, and BRSVMIV in normotensive male offspring with hypertensive parents, along with sympathovagal imbalance, indicate an increased susceptibility to developing hypertension at an earlier age. Evaluating autonomic function and BRS alongside comorbid factors can facilitate the planning of early preventive lifestyle intervention measures with emphasis on physical activity, diet modification, adequate sleep, and stress handling to improve the cardiovascular health of these individuals. The assessment of BRS during various physiological challenges can enhance our understanding of the genesis of hypertension in this high-risk vulnerable population.

Limitations

In this study, we did not categorize normotensive male offspring based on the parental history of hypertension, such as paternal, maternal, or both parents being hypertensive, age of onset, and duration of hypertension in parents, as moderate sample size was a limiting factor. A study conducted on a larger sample size would have allowed for such categorization, providing more insights into the effect of hereditary factors on the onset of hypertension in offspring.

Future perspectives

Future research with a larger sample size should categorize normotensive male offspring based on parental history of hypertension (paternal, maternal, or both parents being hypertensive, age of onset, and duration of hypertension). This would enhance understanding of hereditary factors’ effect on hypertension onset. Comparing male and female offspring may reveal gender-specific differences in baroreceptor sensitivity and other comorbid factors influenced by familial hypertension. Further investigations into the mechanisms linking BRS with metabolic and vascular markers could identify targets for preventive interventions in this high-risk population.

## Conclusions

In conclusion, our study demonstrates reduced baroreflex sensitivity both at rest and while performing the Valsalva maneuver (phase II and IV), increased sympathetic activity, decreased vagal tone, exaggerated diastolic blood pressure response to isometric handgrip, insulin resistance, dyslipidemia, and reduced levels of apelin and relaxin in normotensive male offspring with hypertensive parents. The reduced BRS in these individuals is the result of sympathovagal imbalance, influenced by the presence of multiple comorbid factors, and places them at a heightened risk for future development of hypertension at an early age.
